# Satisfaction of family members of critically ill patients admitted to a public hospital intensive care unit and correlated factors

**DOI:** 10.5935/0103-507X.20190024

**Published:** 2019

**Authors:** Thais Dias Midega, Henrique Souza Barros de Oliveira, Renata Rego Lins Fumis

**Affiliations:** 1 Unidade de Terapia Intensiva, Hospital Geral do Grajaú - São Paulo (SP), Brasil.; 2 Unidade de Terapia Intensiva, Hospital Sírio-Libanês - São Paulo (SP), Brasil.

**Keywords:** Terminal ill, Critical illness, Family, Knowledge, Anxiety, Depression, Consumer behavior, Doente terminal, Estado terminal, Família, Conhecimento, Ansiedade, Depressão, Comportamento do consumidor

## Abstract

**Objective:**

To analyze the satisfaction, medical situation understanding and symptoms of anxiety and depression in family members of patients admitted to the intensive care unit.

**Methods:**

The family members of patients who were hospitalized for ≥ 72 hours were invited to participate in the study, which was performed in a public hospital. Questionnaires were answered to assess the understanding of the diagnosis, treatment and prognosis, and the support received in the intensive care unit. The family needs were also evaluated using a modified version of the Critical Care Family Needs Inventory (CCFNI). The Hospital Anxiety and Depression Scale (HADS) was used to assess the symptoms of anxiety and depression.

**Results:**

A total of 35 family members were interviewed within the patients' first week of stay in the intensive care unit. Most patients (57.1%) were male, aged 54 ± 19 years. Sepsis was the main reason for admission to the intensive care unit (40%); the median of the Simplified Acute Physiology Score (SAPS) 3 was 68 (48 - 77), and 51.4% of the patients died in the intensive care unit. The majority of the family members were female (74.3%) and were sons or daughters of patients (54.3%), with a mean age of 43.2 ± 14 years. Overall, 77.1% of the family members were satisfied with the intensive care unit. A total of 37.1% of the family members did not understand the prognosis. Receiving clear and complete information in the intensive care unit and the doctor being accessible were factors that were significantly correlated with the overall family satisfaction. The prevalence of symptoms of anxiety (60%) and depression (54.3%) in the family members was high.

**Conclusion:**

The emotional distress of family members is high during a patient's hospitalization in the intensive care unit, although satisfaction is also high. Clear and complete information provided by the intensivist and the support received in the intensive care unit are significantly correlated with the satisfaction of family members in a public hospital.

## Abstract (pt)

**Objetivo:**

Analisar a satisfação, a compreensão e os sintomas de ansiedade e depressão em familiares de pacientes admitidos na unidade de terapia intensiva.

**Métodos:**

O familiar do paciente com tempo de internação ≥ 72 horas foi convidado a participar do estudo, realizado em um hospital público. Foram respondidos questionários para avaliar a compreensão do diagnóstico, do tratamento e do prognóstico, e o suporte recebido na unidade de terapia intensiva. Também foram avaliadas as necessidades da família por meio da versão modificada do *Critical Care Family Needs Inventory* (CCFNI) e foi aplicada a *Hospital Anxiety and Depression Scale* (HADS), para avaliar os sintomas de ansiedade e depressão.

**Resultados:**

Foram entrevistados 35 familiares em sua primeira semana de permanência na unidade de terapia intensiva. A maioria dos pacientes (57,1%) era do sexo masculino, com 54 ± 19 anos de idade. A sepse foi o principal motivo da internação na unidade de terapia intensiva (40%); a mediana do *Simplified Acute Physiology Score* (SAPS) 3 foi de 68 (48 - 77) e 51,4% faleceram na unidade de terapia intensiva. A maioria dos familiares era do sexo feminino (74,3%), filhos ou filhas dos pacientes (54,3%), com idade de 43,2 ± 14 anos. Foi observado que 77,1% dos familiares encontravam-se satisfeitos com a unidade de terapia intensiva. A incompreensão do prognóstico foi observada em 37,1% dos familiares. As informações claras e completas recebidas na unidade de terapia intensiva e o médico ser acessível tiveram correlação significativa com a satisfação geral da família. Foi grande a prevalência dos sintomas de ansiedade (60%) e depressão em (54,3%) nos familiares.

**Conclusão:**

O sofrimento emocional dos familiares é grande durante a internação do paciente na unidade de terapia intensiva, embora a satisfação seja alta. As informações claras e completas dadas pelo intensivista e o suporte recebido na unidade de terapia intensiva têm correlação significativa com a satisfação dos familiares em um hospital público.

## INTRODUCTION

Hospitalization in an intensive care unit (ICU) is seen as a crisis situation for the patient and his or her family that can generate anxiety, depression and posttraumatic stress disorder, among other symptoms, that comprise post-ICU syndrome.^(1-5)^

At the end of the 1970s, the pioneering work of Molter identified the needs of the family of the critical patient. Notably, 50% of the ten most important needs were related to communication.^(6)^ Since then, there has been a constant concern for the effectiveness of communication, given its importance.^(7-10)^

Previous reports have identified that families often have difficulties in understanding the diagnosis, treatment and prognosis of their loved one. Studies show that approximately 50% of family members do not understand one of these three factors. The prognosis is the least understood, and failure to understand it is associated with family dissatisfaction.^(11-14)^

Psychological stress has also been the subject of considerable attention in recent years, given that the ICU is one of the most stressful environments of the hospital, and family members often have a high prevalence rate of anxiety and depression symptoms early upon admission to the ICU, which is a risk factor for posttraumatic stress.^(1,3-5,15)^ To make communication more effective, a well-conducted family conference is essential to minimize difficulties and uncertainties and to improve communication between staff and family.^(4,8,9)^ Strategies for this have been established, such as having a conference with the family 72 hours after admission with active listening, respect and compassion; understanding the desires and values of the patient and family; and integrating with communication with palliative care. These measures are reflected in the improved understanding and satisfaction of family members, in good final decisions and in the reduction of symptoms of anxiety, depression and posttraumatic stress.^(4,8,9,16)^ Undoubtedly, the understanding, satisfaction and emotional state of the family are essential, considering the complex ICU environment, and these factors are intertwined.^(4,9,10,15-17)^

The level of satisfaction is quite variable among hospitals. Among private hospitals, which have more resources and more options for meeting the needs of family members, satisfaction is significantly higher compared to that in public hospitals.^(15,18,19)^ According to Freitas et al., satisfaction in public hospitals is mainly influenced by information on the patient's progress.^(18)^

According to previous studies, the information and support received in ICUs are determinants for family satisfaction,^(10,17)^ and effective communication is an important factor to reduce symptoms of anxiety and depression and to prevent posttraumatic stress.^(4,20)^ It is important to detect failures in understanding, the level of satisfaction and symptoms of anxiety and depression in family members at an early stage. Few studies have explored the correlation between these three factors-satisfaction, understanding and symptoms of anxiety and depression-in a public hospital that typically serves people with low income and educational levels.^(18,19)^

The goal of the present study was to analyze the satisfaction, understanding and symptoms of anxiety and depression in family members of patients admitted to a public hospital ICU.

## METHODS

This was a longitudinal study conducted in the ICU of the Hospital Geral do Grajaú (HGG), São Paulo. For patients admitted to the HGG ICU with hospitalization times ≥ 72 hours, the immediate family member who was responsible for the patient (spouse, child, parent or sibling) was invited to participate in this study. After signing the informed consent form, the family member responded to the questionnaire shortly after the visitation time and after receiving medical information, without the presence of any other person. The questionnaire was filled out by the researcher.

This study was approved by the Ethics and Research Committee of the *Instituto de Responsabilidade Social Sírio-Libanês*, CAAE 66572317.7.1001.5447, opinion 2.028.802, dated April 24, 2017.

Only one family member per patient was invited to participate in the study. The patient had to be in the ICU for at least 72 hours; the family member had to be older than 18 years, be an immediate family member of the patient (spouse, parent, child or sibling) and be responsible for maintaining contact with the doctor and visiting the patient in the ICU.

The following family members were excluded from the study: family members of patients who were dying, with a probability of death in less than 48 hours; those who were not able to understand the questions of the questionnaire; those who were not an immediate family member of the patient; and those who were not present to receive information from the ICU medical team at the time of visitation.

The HGG has two ICUs (North and South units), and the study was conducted only in the South unit, which contains the most severe patients. The health professional: patient ratios at the time of the study were as follows: nurse 1:8; nursing technician 1:2; and physician 1:4.

The ICU visitation policy allowed for two visitation periods: one in the afternoon from 3:00 pm to 4:00 pm and the other at night from 8:00 pm to 9:00 pm. In the afternoon, the medical team talked with the family about the clinical status of the patient; at night no further information was provided. Whenever the medical team decided that the presence of the family was necessary, as in cases of probable death, the visitation periods were flexible, and the family could stay up to 24 hours.

Daily, during the visit, the researcher identified the family member who met the inclusion criteria and asked him or her to participate in the study. When the family member consented to be included in the study, the questionnaire was answered after he or she received medical information in the ICU. The questionnaire was administered by the main researcher, and the family member was told that his or her name would be kept confidential.

After interviewing the family member, the researcher asked the physician in charge questions about the diagnosis, treatment, prognosis and procedures performed to check whether the answers given by the family member were correct or not.

### Tools

To assess the family needs, the modified version of the *Critical Care Family Needs Inventory* (CCFNI) instrument was used, with the text translated and validated by Fumis et al.^(12)^ This instrument consists of 14 questions, with responses varying from "almost always" to "never". To assess satisfaction (needs found), a cutoff point of 9 was used, according to Fumis et al.^(10,12)^

An assessment of the family member's understanding of the diagnosis, treatment, prognosis and procedures was performed according to the criteria used by Azoulay^(11)^ and Fumis.^(12)^

The understanding of the diagnosis was defined according to the main reason for ICU admission, considering a list with names that are easy for laypeople to understand (e.g., heart, respiratory problems, neurological problems, trauma, renal); whether the treatment was clinical or surgical; and whether the prognosis corresponded to the correct expectation regarding the outcome in the ICU, namely, "severe, patient survival not expected"; "severe, but with expected survival of the patient" and "not severe, patient survival is expected." This study did not intend to measure specific knowledge on issues discussed with the intensive care physician regarding the prognosis. The family member's understanding was compared with the actual ICU outcome (discharge or death), generating a dichotomous variable (right or wrong understanding of the severity of the patient).

The family member marked which procedures were performed in the ICU up to the time of the interview from among a list of ten possible procedures: tracheostomy, mechanical ventilation (intubation), aspiration of tracheal or naso-tracheal cannula, noninvasive mechanical ventilation (facial or nasal mask), deep vein catheter, urinary catheter, chest drainage, surgical drainage, hemodialysis, and cardiac monitoring.

The evaluation of satisfaction with the support received at the ICU regarding information and care was also performed according to the criterion used by Fumis.^(10)^

To evaluate the symptoms of anxiety and depression, the *Hospital Anxiety and Depression Scale* (HADS) was used, with a cutoff of > 10 points for each subscale, which was used in previous studies.^(1,3,15)^

### Statistical analysis

Continuous data were expressed as measures of central tendency and dispersion measurements. Categorical data were expressed as absolute (n) and relative (%) frequency distributions. Kendall's coefficient of concordance and the Spearman association test were used to assess the concordance between the level of information received by the family in the ICU, the level of support received, and the overall satisfaction with the ICU. A two-sided p-value ≤ 0.05 was considered statistically significant. The software Statistical Package for Social Sciences (SPSS) version 20.0 from IBM^®^ was used to perform the analyses.

## RESULTS

Between October 2017 and March 2018, 86 patients remained in the HGG ICU for ≥ 72 hours. Of these, 39 were excluded as follows: 28 due to the probability of death occurring in less than 48 hours; 11 family members were unable to understand the questionnaire; and there were 12 refusals to participate in the study. Thirty-five family members of patients admitted to the ICU for ≥ 72 hours participated in the study. The family members of critical patients were interviewed at a median of 5 (3 - 8) days after admission to the ICU, with a median stay in the ICU of 14 (8 - 21) days.

### Patient characteristics

Regarding the patients, 20 (57.1%) were male, and the majority declared being married (51.4%). The age ranged from 18 to 83 years old, with a mean of 54 ± 19 years. Regarding the origin of the hospitalization, 22 (62.9%) were from the emergency room and 7 (20%) were from the shock unit. Regarding the type of outcome, 18 (51.4%) died and 17 (48.6%) were discharged (Table 1).

**Table 1:** Sociodemographic and clinical characteristics of patients

Variables	Values
Patients	35 (100)
Age (years)	54.5 ± 19 (18 - 83)
Gender	
Male	20 (57.1)
Female	15 (42.9)
Marital status	
Single	7 (20.0)
Married	18 (51.4)
Widowed	5 (14.3)
Divorced	5 (14.3)
Length of hospitalization (days)	14 [8 - 21] (4 - 66)
Type of treatment	
Clinical	29 (82.9)
Surgical	6 (17.1)
Reason for hospitalization	
Heart Diseases	5 (14.3)
Pneumopathies	3 (8.6)
Neuropathies	4 (11.4)
Trauma	4 (11.4)
Renal diseases	2 (5.7)
Gastroenteropathy	3 (8.6)
Sepsis	14 (40)
SAPS 3	68 [48 - 77]
Origin of the hospitalization	
Emergency	22 (62.9)
Shock unit	7 (20)
Ward	6 (17.1)
Outcome	
Discharge	17 (48.6)
Death	18 (51.4)

SAPS - Simplified Acute Physiology Score. Results expressed as n (%), mean ± standard deviation, median [interquartile] (variation) or median [interquartile range].

The predominant reason for ICU admission was sepsis (40%); 85.7% of the patients received a severe medical prognosis but with expected survival. The Simplified Acute Physiology Score (SAPS) 3 showed a median score of 68 (48 - 77) points.

### Family member characteristics

The majority of family members were female (26; 74.3%), with a mean age of 43.2 ± 14 years, with the sample varying between 18 and 73 years. Regarding the level of education, 24 (68.6%) had completed high school. In terms of religion, 19 (57.3%) were Catholic and 12 (34.3%) were Protestant. Regarding their relationship to the ICU patients, most of the patients were their child (54.3%), and approximately half of the family members had previous experience in this or another ICU (54.3%) (Table 2).

**Table 2:** Characteristics of family members

Variables	Values
Family Members	35 (100)
Age (years)	43.2 ± 14.9 (18 - 78)
Gender	
Female	26 (74.3)
Marital status	
Single	10 (28.6)
Married	21 (60)
Widowed	3 (8.6)
Divorced	1 (2.9)
Education	
Elementary School	13 (37.1)
High School	11 (31.4)
Higher Education	11 (31.4)
Religion	
Catholic	19 (54.3)
Evangelical	12 (34.3)
Other	4 (1.5)
Degree of relationship	
Spouse	05 (14.3)
Parent	06 (17.1)
Child	19 (54.3)
Sibling	5 (14)
Prior ICU experience	
I have never been to an ICU	16 (45.7)

ICU - intensive care unit. Results expressed by n (%), mean minimum-maximum.

### Level of family members' satisfaction and understanding

A total of 22.9% of the family members were dissatisfied with the ICU. The assessment with the overall satisfaction with the ICU was scored on a scale of 1 to 14, and the median score was 11 (10 - 13).

Regarding understanding, only 2.9% of family members did not understand the real reason for ICU admission, and 5.7% did not understand the treatment. The greatest difficulty found was related to the prognosis; 37.1% of the family members had erroneous expectations regarding the ICU outcome, and 25.7% of the family members had disagreements regarding the medical expectations. Regarding procedures that were less well-understood, i.e., those for which the family could not answer whether they had or had not been performed, the greatest difficulty was in relation to deep vein catheter (23, 65.7%), followed by urinary catheterization (42, 9%), cardiac monitoring (12, 34.3%) and intubation (4, 11.4%) (Table 3).

**Table 3:** Understanding the family members of critically ill patients regarding the procedures performed in the intensive care unit

Procedures	Total performed n = 35	Correct answers n (%)	Incorrect answers n (%)
Tracheostomy	2 (5.7)	35 (100)	0 (0.0)
Aspiration	26 (74.3)	21 (60)	14 (40)
Intubation	28 (80)	31 (88.6)	4 (11.4)
NIMV	6 (17.1)	29 (82.9)	06 (17.1)
Deep vein catheter	32 (91.4)	12 (34.3)	23 (65.7)
Thoracic drainage	02 (5.7)	32 (91.4)	3 (8.6)
Bladder catheter	34 (97.1)	20 (57.1)	15 (42.9)
Surgical drain	4 (11.4)	33 (94.3)	2 (5.7)
Hemodialysis	11 (31.4)	33 (94.3)	2 (5.7)
Cardiac monitoring	35 (100)	23 (65.7)	12 (34.3)

NIMV - noninvasive mechanical ventilation. A correct answer refers to the correct answers of the family, regardless whether the procedure was performed; this is the same for incorrect answers.

### Associations between information and support received in the intensive care unit and overall family satisfaction

Table 4 shows some of the associations made between the information and support received in the ICU versus overall family satisfaction. We observed significant associations between the family satisfaction in the intensive care setting and the information given by the ICU physician (R: 0.556; p = 0.001), mainly regarding the diagnosis at admission (R: 0.660; p < 0.0001), the causes (R: 0.475; p = 0.004) and the consequences of the disease (R: 0.665; p < 0.001).

**Table 4:** Significant associations between information and support received in the intensive care unit and overall family satisfaction

	Degree of agreement with overall satisfaction (> 9 points) n (%)		Kendall	p value
Information received				
Regarding the diagnosis of admission to ICU	Satisfied	26 (74.3)	0.660	< 0.0001
	Dissatisfied	4 (11.4)		
Regarding the causes of the disease	Satisfied	25 (71.4)	0.475	0.004
	Dissatisfied	4 (11.4)		
Regarding the consequences of the disease	Satisfied	26 (74.3)	0.665	< 0.0001
	Dissatisfied	5 (14.3)		
Given by ICU physicians	Satisfied	26 (74.3)	0.556	0.001
	Dissatisfied	4 (11.4)		
Support received at the ICU				
The ICU doctor was sympathetic	Satisfied	27 (77.1)	0.452	0.006
	Dissatisfied	2 (5.7)		
The ICU doctor was accessible	Satisfied	25 (71.4)	0.578	< 0.0001
	Dissatisfied	5 (14.3)		

ICU - intensive care unit.

In addition, there were also significant associations between the support given to family members and the level of satisfaction, and the most important factors for family members' satisfaction occurred when the intensive care physician was accessible (R: 0.578; p < 0.0001) and easy to understand (R: 0.452; p = 0.006) (Table 4).

There were no significant associations between the clinical severity of patients (SAPS 3) and the satisfaction of family members with the ICU (R: -0.147; p = 0.400).

### Symptoms of anxiety and depression in family members during intensive care unit hospitalization

There was a high prevalence of symptoms of anxiety (60%) and depression (54.3%) among family members. The total HADS score had a median of 24 (17 - 31) points. The median HADS anxiety subscale score was 12 (9 - 17) points, and the HADS depression subscale score was 12 (5 - 23) points. There was a correlation between anxiety and depression symptoms and the risk of death, i.e., when the medical prognosis was severe, with the unexpected survival of the patient, there were higher scores on the HADS scale of anxiety and depression symptoms (R = 0.432; p = 0.010). There was an association regarding the risk of death and actual death (likelihood ratio p = 0.032). There was no association between depression and anxiety symptoms and the level of satisfaction.

## DISCUSSION

In a complex ICU setting, the satisfaction, comprehension and emotional state (symptoms of anxiety and depression) of family members are essential factors in the assessment of care given to a family. Humane treatment in ICUs requires empathetic communication, respect and compassion, and warmth in shared decisions with family members in an attempt to reduce suffering; occasionally this suffering extends beyond the ICU and is called post-ICU syndrome, i.e., posttraumatic stress, symptoms of anxiety and depression and complicated grief.^(4,5,7-9,20)^

The present study aimed to analyze the symptoms of anxiety and depression, the understanding and the satisfaction of family members of critically ill patients in a public hospital ICU. A high level of family member satisfaction was observed and was similar to that reported in the private hospital network. However, our population has a lower educational level, which contributes to greater satisfaction, perhaps because these individuals are less demanding.^(12,19)^

It is very important to the family to receive clear and complete information and to feel that the ICU doctor is accessible and understanding. In previous studies, conflicting and incomplete information on the causes and consequences of the disease, in addition to doctor inaccessibility, were determinants for dissatisfaction.^(10,17)^

However, despite the high satisfaction level, the prevalence of anxiety and depression symptoms was well above that reported in the literature. Greater than 50% of the family members had these symptoms in the first week after patient admission to the ICU. In agreement with other studies, some factors may have impacted the emotional state of the family, such as a younger patient age; the severity level, with very high SAPS 3; a high frequency of sepsis; and a very high mortality in this ICU. ^(1,3,15,21,22)^

Furthermore, we detected a high difficulty in understanding the prognosis. It is necessary to draw attention to this recurrent difficulty, which has been shown in the literature.^(11-14)^ Understanding the prognosis is undoubtedly the most difficult element due to fluctuations in a patient's status, and understanding the prognosis is also a difficulty that is observed among physicians. However, because understanding the prognosis is an element associated with satisfaction and often causes disagreement, it needs to be better understood, and failing to do so will always cause concern.^(10,12,14)^

It has been shown that open visitation policies allow the patient to benefit from family support; however, this type of policy remains rare in Brazil and worldwide. With open visitation policies, communication becomes more effective and the satisfaction of family members increases.^(15,23-28)^ In Brazil, ICUs with a 24-hour open visitation policy are rare (2.6%).^(23)^ The HGG ICU is typical of most Brazilian ICUs, where 45.1% of ICUs allow two daily visitation periods, and 69.1% of ICUs allow 30 to 60 minutes of visitation time per period. In addition, in special situations, such as end-of-life cases, 98.7% of ICUs allow visits at flexible times.^(23)^ According to a recent review, flexibility visitation provides remarkable benefits, such as the reduced delirium of patients, a reduction of anxiety and depression symptoms and the improvement of family satisfaction, possibly due to the greater contact of family members with the team of health professionals, which facilitates communication and accessibility.^(24,25)^

Some factors can impact both the patient and the family. In the ICU studied here, the patient beds are separated by curtains and, according to previous studies, this is a risk factor for family member depression and for patient delirium.^(3,24)^ In addition, there is no waiting room near the HGG ICU, which was previously found to be associated with dissatisfaction and depression symptoms and is a major need for the family.^(6,17,29)^

Consistent with previous studies, family satisfaction does not depend on the outcome of the patient. Given the worst outcome in the ICU, the main determinants for satisfaction are the effective communication and support received in the ICU.^(30)^ Warm and effective communication and good decision-making are important to meet the needs of the family in difficult times. It is necessary to devote more time to listening to the family; to provide complete and clear information in the ICU; to demonstrate respect, compassion and empathy regarding the feelings and beliefs of the family; to be accessible and welcoming; and not to use technical and sophisticated terms when talking with the family.^(4,9,10,12,31)^

Regarding the procedures performed in the ICU, the family members did not understand the simplest procedures, such as cardiac monitoring, or the more invasive ones, such as peripheral vein and urinary catheters. It is important to verify the knowledge gaps of family members to keep them informed of the procedures performed in their absence, especially in the case of invasive procedures, which may be associated with infectious conditions and changes in the patient's condition.^(32,33)^

A high prevalence of anxiety and depression symptoms was observed among family members of patients who were severely ill and those at high risk of mortality. It is important to emphasize that the interview occurred prior to the death of the patient, at the beginning of the ICU stay. However, we can assume that the high prevalence of anxiety and depression symptoms is due to the perception of an unfavorable outcome and considering that a high severity level in younger patients is a risk factor for anxiety and depression symptoms, as expected.^(1,3,19,20)^ Another important consideration is the predominance of female family members in this sample, and being female is a major risk factor for the development of symptoms of anxiety and depression.^(34)^

This study has major limitations. The study included a small number of family members from a single center and, therefore, its conclusions need to be interpreted with caution. In addition, there was no information available on the presence of symptoms of anxiety and depression prior to admission to the ICU or other psychosocial factors that could have affected the sample. Finally, the questionnaires were administered in the first week, and we do not know whether family member satisfaction decreased over time. Additionally, the incidence of anxiety and depression symptoms increases with the length of ICU hospitalization.

The present study emphasizes the importance of welcoming patients and providing quality information to those who have a loved one in the ICU, which is a very stressful environment. It is possible to meet family needs in the public health system, but it is necessary to do more, such as offering psychological support in the ICU in an attempt to reduce the high prevalence of anxiety and depression symptoms, especially in the case of family members of severely ill patients.

## CONCLUSION

This study suggests that the medical team should be accessible and sympathetic and provide complete information on the diagnosis at admission and information regarding the causes and consequences of the disease. This information is important for those who accompany their loved ones to the intensive care unit and is associated with greater satisfaction. In addition, we emphasize the need for psychological support in the intensive care unit due to the high prevalence of anxiety and depression symptoms in family members, especially those of patients with poor prognoses.
